# Structural basis for BIR1-mediated negative regulation of plant immunity

**DOI:** 10.1038/cr.2017.123

**Published:** 2017-09-29

**Authors:** Cuiyan Ma, Yanan Liu, Bing Bai, Zhifu Han, Jiao Tang, Heqiao Zhang, Hoda Yaghmaiean, Yuelin Zhang, Jijie Chai

**Affiliations:** 1 School of Life Sciences, Peking University, Beijing 100871, China; 2 Department of Botany, University of British Columbia, Vancouver, BC V6T 1Z4, Canada; 3 Ministry of Education Key Laboratory of Protein Science, Center for Structural Biology, School of Life Sciences, Tsinghua-Peking Joint Center for Life Sciences, Tsinghua University, Beijing 100084, China; 4 Institute of Apicultural Research, Chinese Academy of Agricultural Sciences, Beijing 100093, China

## Dear Editor,

Plant receptor kinases (RKs) can function as pattern recognition receptors (PRRs) for perception of pathogen-associated molecular patterns (PAMPs) to induce immune responses^1,2^. One of such PRRs is the leucine-rich repeat RK (LRR-RK) FLAGELLIN-SENSING 2 (FLS2) that recognizes bacteria-derived flagellin (flg22 epitope)^3,4^. The smaller LRR-RK BRI1-associated kinase 1 (BAK1) acts as a co-receptor with FLS2^5,6^.

The *Arabidopsis* LRR-RK BAK1-interacting receptor-like kinase 1 (BIR1) was initially identified through a reverse genetic screen and the phenotypes of *bir1*-*1* can be suppressed by the adapter LRR-RK SOBIR1 (suppressor of BIR1)^7^. SOBIR1 interacted with BAK1 *in planta* when the expression of BIR1 was silenced, suggesting that BIR1 sequesters BAK1 from SOBIR1 in resting cells to inhibit cell death and immune responses^8^. All four BIR members (BIR1-BIR4) in *Arabidopsis* interacted with BAK1 when expressed in *Nicotiana benthamiana*. Like *bir1*, *BAK1-interacting receptor-like kinase 2* (*bir2*) mutants also display enhanced SA-dependent cell-death^9^. BIR2 also has a critical role in negative regulation of flg22-induced responses by controlling BAK1-FLS2 complex formation in a ligand-dependent manner^9^.

**Figure 1:**
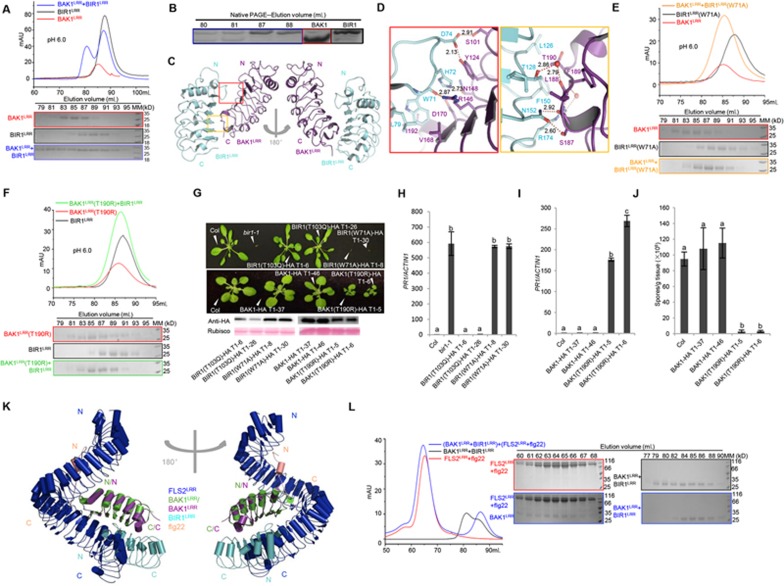
Interaction between BAK1^LRR^ and BIR1^LRR^ is required for BIR1 inhibition of BAK1-mediated plant immunity. **(A)** Gel filtration profiles of BAK1^LRR^ and BIR1^LRR^ at pH 6.0. The vertical and horizontal axes represent ultraviolet absorbance (λ = 280 nm) and elution volume (mL), respectively. Bottom panel, coomassie blue staining of the peak fractions following SDS-PAGE. The numbers shown on the top of the SDS-PAGE gels indicate elution volumes (mL). Frame colors of the SDS-PAGE gels are equivalents to those of the gel filtration profiles for proteins indicated. MM: molecular weight maker. Hiload 200 was used for the gel filtration assays. **(B)** Native-PAGE coomassie blue staining of the peak fractions for the gel filtration at pH 6.0 in **(A)**. **(C)** Overall structure of the BAK1^LRR^-BIR1^LRR^ complex. “N” and “C” represent the N- and C-terminus, respectively. **(D)** Detail interactions between BAK1^LRR^ and BIR1^LRR^. The left panel, the interface between the N-terminal side of BIR1^LRR^ and BAK1^LRR^. The right panel, the interface between the C-terminal portion of BAK1^LRR^ and BIR1^LRR^. Red dashed lines indicate polar interactions and their distances are labeled. T, Thr; L, Leu; N, Asn; S, Ser; Y, Tyr; D, Asp; H, His; R, Arg; W, Trp; V, Val; I, Ile; F, Phe. **(E)** Mutagenesis analysis of the BAK1^LRR^-BIR1^LRR^ (W71A) complex using gel filtration. **(F)** Mutagenesis analysis of the BAK1^LRR^ (T190R)-BIR1^LRR^ complex using gel filtration. **(E** and **F)** The assays were performed as described in **(A)**. **(G)** Morphological phenotypes of transgenic plants expressing the BIR1 (T103Q) -HA or BIR1 (W71A)-HA protein in *bir1-1*, and the BAK1-HA or BAK1 (T190R)-HA protein under its native promoter in wild type background (Col-0). The photograph shows four-week-old soil-grown plants. Expression of proteins was detected by western blot using an anti-HA antibody. **(H** and **I)** Expression levels of *PR1* in the indicated genotypes as determined by quantitative RT-PCR. Two-week-old seedlings grown on ½ MS plates were used for the assays. Values were normalized to the expression levels of *ACTIN1*. The data are shown as means ± SD (*n* = 3) with one-way ANOVA and Tukey's test. Different letters indicate significant differences (*P* < 0.01). The experiments were repeated three times with similar results. **(J)** Growth of *H. a.* Noco2 on seedlings of the indicated genotypes. The data are shown as mean ± SD (*n* = 3) with one-way ANOVA and Tukey's test. Different letters indicate significant differences (*P* < 0.01). The experiments were repeated three times with similar results. **(K)** Structural superimposition of the BAK1^LRR^-BIR1^LRR^ complex with that of FLS2^LRR^-flg22-BAK1^LRR^ using BAK1^LRR^ as the template. “N” and “C” represent the N- and C-terminus, respectively. Color codes are indicated. This alignment was performed by the program COOT. **(L)** FLS2^LRR^-flg22 releases BAK1^LRR^ from the BAK1^LRR^-BIR1^LRR^ complex in gel filtration at pH 6.0. BIR1^LRR^ and BAK1^LRR^ with a molar ratio of about 2.5:1 were mixed together and incubated at 4 °C for 30 min. The FLS2^LRR^-flg22 complex was then added to the mixture for gel filtration. The molar ratio between FLS2^LRR^ and BAK1^LRR^ was about 2:1. Shown in the left panel are gel filtration profiles of proteins indicated. The vertical and horizontal axes represent ultraviolet absorbance (λ = 280 nm) and elution volumes (mL), respectively. Right panel, coomassie blue staining of the peak fractions following SDS-PAGE. The numbers shown on the top of the gels indicate elution volumes (mL). MM: molecular weight maker.

We first examined BAK1-BIR1 interaction using their extracellular LRR portions expressed in insect cells. BIR1^LRR^ and BAK1^LRR^ formed a stable heterodimeric complex at pH 6.0 in gel filtration (Figure 1A, Supplementary information, Figure S1), which was further confirmed by native gel analysis of the gel filtration fractions (Figure 1B). The BAK1^LRR^-BIR1^LRR^ interaction was further supported by Isothermal Titration Calorimetry (ITC) and Sedimentation-Velocity Analytical UltraCentrifugation (SV-AUC) analyses (Supplementary information, Figure S2). At pH 4.0, BAK1^LRR^ and BIR1^LRR^ still interacted with each other (Supplementary information, Figure S3A) in gel filtration. However, BAK1^LRR^ lost its activity of interacting with BIR1^LRR^ at pH 8.0 (Supplementary information, Figure S3B). These results indicate that the BAK1^LRR^-BIR1^LRR^ interaction *in vitro* is pH-dependent, which is further supported by the ITC data (Supplementary information, Figure S4). Similar to BIR1^LRR^, BIR3^LRR^ and BIR4^LRR^ also displayed interaction with BAK1^LRR^ at pH 6.0 (Supplementary information, Figure S5A, S5C, S5D). But unlike BIR1^LRR^, BIR3^LRR^ and BIR4^LRR^ still interacted with BAK1^LRR^ at pH 8.0 in both gel filtration and ITC assays (Supplementary information, Figure S6A, S6C, S6D). Compared to BIR1^LRR^, BIR3^LRR^ and BIR4^LRR^, BIR2^LRR^ exhibited a much weaker affinity towards BAK1^LRR^ at pH 6.0 (Supplementary information, Figure S5A, S5B). Like BIR1^LRR^, BIR2^LRR^ also had no detectable interaction with BAK1^LRR^ at pH 8.0 in gel filtration and ITC assays (Supplementary information, Figure S6A, S6B). These biochemical data indicate that BIR1-4 and BAK1 directly interact with each other through their ecto-domains.

To probe the molecular mechanism underlying BIR^LRR^-BAK1^LRR^ interaction, we solved the crystal structure of the complex (Figure 1C). Interaction between the two proteins is mediated by packing of one lateral side of BIR1^LRR^ against the C-terminal inner surface and the C-terminal capping domain of BAK1^LRR^(Figure 1C). Specifically, a loop region from the N-terminal capping domain of BIR1^LRR^ makes extensive contacts with BAK1^LRR^(Figure 1C). While the overall structure of BIR1^LRR^ remarkably resembles that of BAK1^LRR^ (Supplementary information, Figure S7A), the loop regions from these two proteins are strikingly different in their conformations and primary sequences (Supplementary information, Figure S7).

The BIR1^LRR^-BAK1^LRR^ interaction is mediated by both polar and hydrophobic contacts, and can be divided into two interfaces (Figure 1C). One is mainly mediated by packing of the loop region from the N-terminal capping domain of BIR1^LRR^ against the C-terminal inner surface of BAK1^LRR^. Interaction between one lateral side of BIR1^LRR^ and one short helix of BAK1^LRR^ forms the other interface (Figure 1D). Trp71 of BIR1^LRR^ from the first interface forms extensive interactions with BAK1^LRR^ by being sandwiched by Val168, Asp170 and Ile192 of BAK1^LRR^(Figure 1D, Supplementary information, Figure S8). Several hydrogen bonds also contribute to the interaction around this interface. At the other interface, Thr190 positioned immediately underneath the C-terminal capping domain of BAK1^LRR^ tightly stacks against Phe150 of BIR1^LRR^, and Thr128 of BIR1^LRR^ and Leu188 of BAK1^LRR^ forms a water-mediated hydrogen bond (Figure 1D and Supplementary information, Figure S8).

To confirm our structural observations, we chose two residues from the centers and two from the peripheries of the BAK1^LRR^-BIR1^LRR^ interfaces for mutagenesis analyses. In support of the structure, mutating Trp71 in BIR1 to the smaller alanine residue resulted in loss of interaction with BAK1^LRR^ (Figure 1E, Supplementary information, Figure S9A). Mutations in the equivalent residues in BIR2^LRR^ (W73), BIR3^LRR^ (W67) and BIR4^LRR^ (W60) caused similar effects on their interaction with BAK1^LRR^ (Supplementary information, Figure S10). Similarly, mutating Thr190 in BAK1 to arginine (Figure 1F, Supplementary information, Figure S9B) and His72 in BIR1 to asparagine (Supplementary information, Figure S11) also resulted in no detectable BAK1^LRR^-BIR1^LRR^ interaction. In contrast, mutating Val168 in BAK1 to arginine compromised but did not abolish the BAK1^LRR^-BIR1^LRR^ interaction as indicated by the results of gel filtration and ITC assays (Supplementary information, Figure S12).

We then used the luciferase (LUC) complementation assay to test the effect of the above mutations on BIR1-BAK1 interaction in *N. benthamiana*. Co-infiltration of Agrobacteria containing BAK1-CLuc and BIR1-NLuc, resulted in strong LUC activity (Supplementary information, Figure S13A). In support of our biochemical data, the BIR1 W71A and BAK1 T190R mutations greatly reduced the LUC activity generation (Supplementary information, Figure S13B, S13C). In contrast, the BAK1 V168R and BIR1 H72N mutations only modestly affected the LUC activity (Supplementary information, Figure S13D, S13E). The positive control BIR1 T103Q mutation, found in other BIR proteins (Supplementary information, Figure S14) and located between the two BAK1-BIR1 interfaces (Supplementary information, Figure S8B), did not impact BIR1 interaction with BAK1 in the assay (Supplementary information, Figure S13A). The reduced interaction was not caused by difference in protein levels as the wild type and mutant BAK1-CLuc and BIR1-NLuc proteins were expressed at comparable levels (Supplementary information, Figure S13F).

We then generated transgenic plants and examined their defense responses. The *bir1-1::BIR1 (W71A)* but not the *bir1-1::BIR1 (T103Q)* transgenic plants displayed seeding lethality phenotype, phenocopying the *bir1-1* mutant (Figure 1G). Furthermore, expression levels of pathogenesis-related genes *PR1* and *PR2* were upregulated in *bir1-1::BIR1 (W71A)* but not in *bir1-1::BIR1 (T103Q)* plants (Figure 1H, Supplementary information, Figure S15A). Supporting our biochemical and cell-based assays, expression of *BAK1 (T190R)* but not wild type *BAK1* or *BAK1 (V168R)* led to plant dwarfism, constitutive expression of *PR1* and *PR2* and increased resistance to *Hyaloperonospora parasitica* Noco2 (Figure 1G, 1I, 1J, Supplementary information, Figures S15B and S16). These results suggest that defense responses were constitutively activated in the *BAK1 (T190R)* transgenic plants. Different from the *bir1-1::BIR1 (W71A)* plants, the *bir1-1::BIR1 (H72N)* plants had wild type morphology, did not constitutively express *PR* genes, and were fully susceptible to the virulent oomycete pathogen *Hyaloperonospora parasitica* Noco2 (Supplementary information, Figure S17). The precise reason for the discrepancy between these functional data and the *in vitro* biochemical data remains unclear.

Structural comparison between BAK1^LRR^-BIR1^LRR^ and FLS2^LRR^-flg22-BAK1^LRR^ showed that the C-terminal portion of FLS2^LRR^, which interacts with BAK1^LRR^, completely overlaps with BIR1^LRR^(Figure 1K), suggesting that the flg22-bound FLS2 may compete with BIR1^LRR^ to release BAK1^LRR^ from the BIR1^LRR^-BAK1^LRR^ complex. Supporting this hypothesis, BAK1^LRR^ from the pre-incubated BAK1^LRR^-BIR1^LRR^ complex formed a stable interaction with FLS2^LRR^-flg22 at pH 6.0 (Figure 1L), indicating that the BAK1^LRR^-BIR1^LRR^ interaction had been outcompeted by FLS2^LRR^-flg22.

Our study shows that the ecto-domains of BIR1 and BAK1 are sufficient for them to interact with each other *in vitro* and the interaction is critical to the inhibition of BAK1 function by BIR1. Consistently, over-expression of the ecto-domain together with the trans-membrane segment of BAK1 in plants phenocopies the *BAK1*-overexpressing phenotypes, presumably through sequestering BIR1^10^. Our primary sequence analysis (Supplementary information, Figure S14) suggests that all 4 BIR members may share a common mechanism of interacting with BAK1. We also showed that the flg22-bound FLS2 outcompeted BIR1^LRR^ for binding to BAK1^LRR^, even with an excess of BIR1^LRR^, explaining the observation that *BIR1* has no effect on FLS2-mediated immune responses^7^. Release of the BIR1-sequestered BAK1 by the flg22-bound FLS2 could become easier by an increase in pH during flg22-induced plant immunity. BIR1 has likely evolved to keep BAK1-mediated cell death signaling under tight control, thus preventing undesired autoimmunity. The observation that the ecto-domain of BIR1 is sufficient for inhibition of BAK1 suggests that the signal relieving BIR1-mediated inhibition of BAK1, if present, comes from the extracellular space.

## Supplementary Material

Supplementary information Supplementary information, Figure S1–S17 and Data S1 Materials and Methods
