# Supplementary material for: Structural basis for BIR1-mediated negative regulation of plant immunity
Source: Cell Res. 2017 Sep 29;27(12):1521–4. doi: 10.1038/cr.2017.123 (PMC5717402; doi:10.1038/cr.2017.123)
Supplement: Supplementary information — , Figure S1–S17 and Data S1 Materials and Methods [file cr2017123x1.pdf]

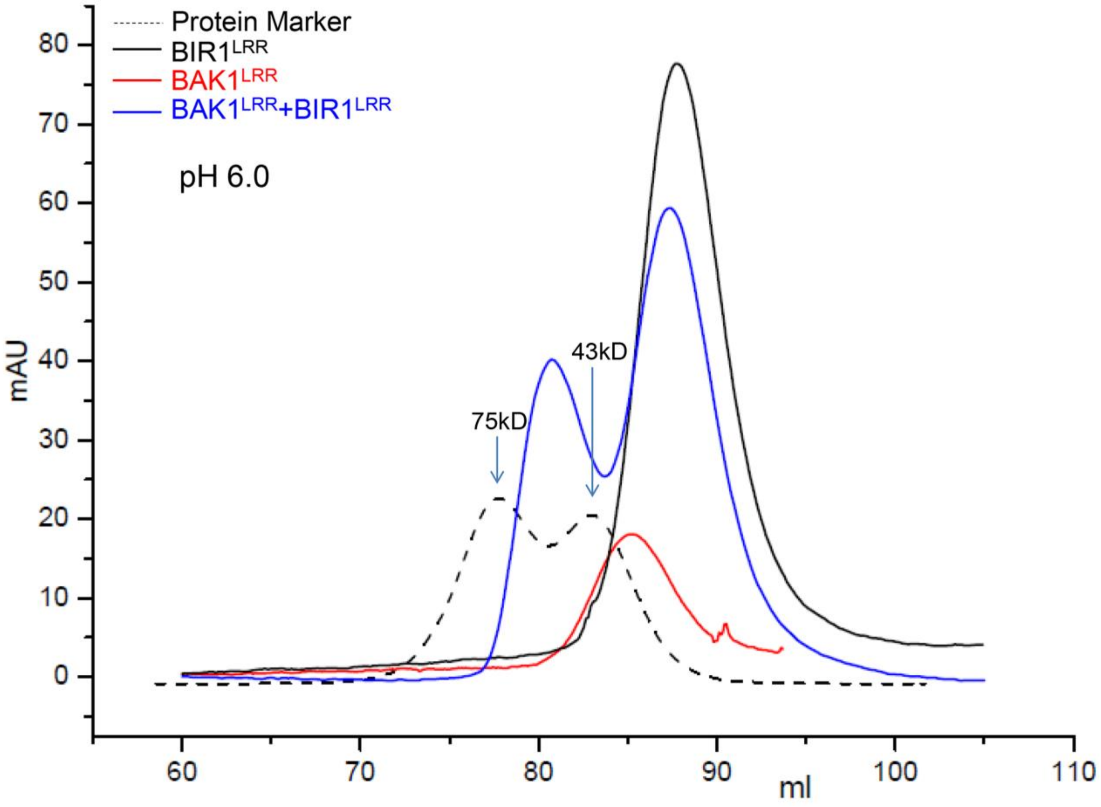

Supplementary information, Figure S1 Gel filtration profiles of protein markers, BAK1^{LRR} and BIR1^{LRR} at pH 6.0. The assays were performed as described in Figure 1**A**.

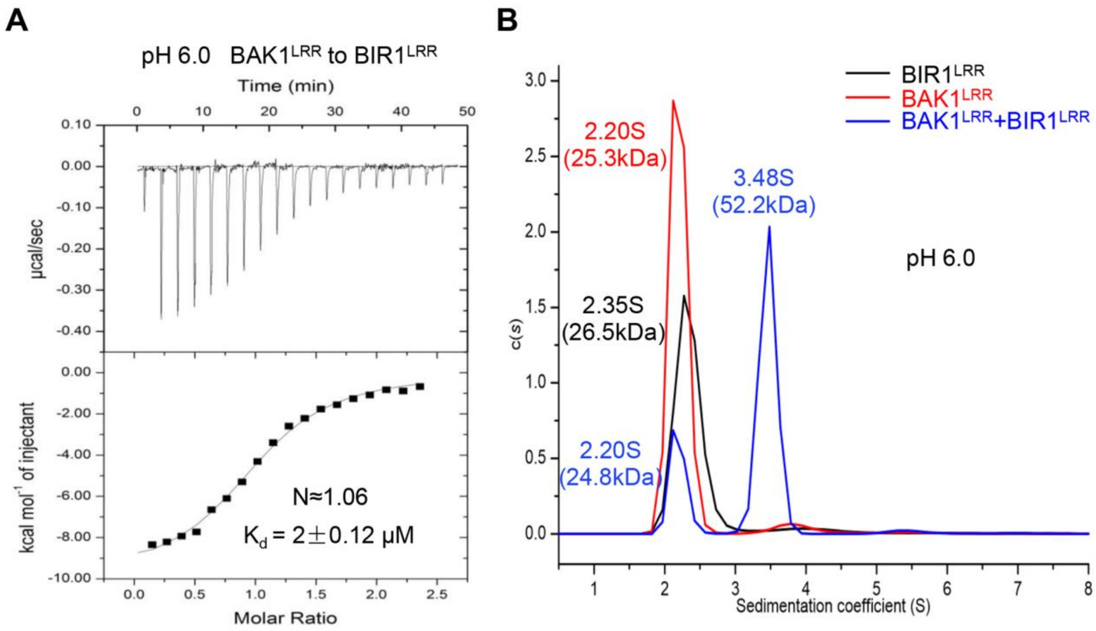

Supplementary information, Figure S2 The interaction between BAK1^{LRR} and BIR1^{LRR} by ITC and sedimentation-velocity analytical ultracentrifugation at pH 6.0.

(A) Measurement of the binding affinity between BAK1^{LRR} and BIR1^{LRR} by ITC at pH 6.0. Top panel: nineteen injections of BAK1^{LRR} solution were titrated into BIR1^{LRR} solution in the ITC cell. The area of each injection peak corresponds to the total heat released for that injection. Bottom panel: the binding isotherm for BAK1^{LRR} and BIR1^{LRR} interaction. The integrated heat is plotted against the molar ratio between BAK1^{LRR} and BIR1^{LRR}. Data fitting revealed a binding affinity of about 2 μM.

(B) Formation of the BAK1^{LRR}-BIR1^{LRR} complex in sedimentation-velocity analytical ultracentrifugation at pH 6.0. The peak sedimentation coefficients and the calculated molecular weights for the proteins indicated are shown.

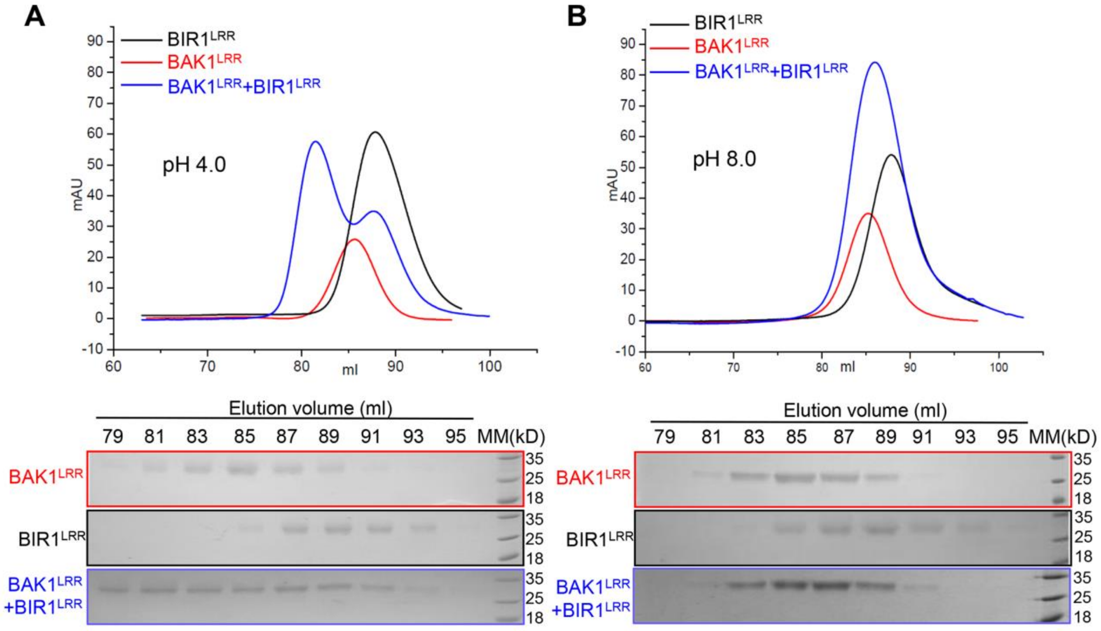

Supplementary information, Figure S3 The interaction between BAK1^{LRR} and BIR1^{LRR} by gel filtration at pH 4.0 and pH 8.0.

The assays were performed as described in Figure 1**A**, except that buffer of pH 4.0 and pH 8.0 were used.

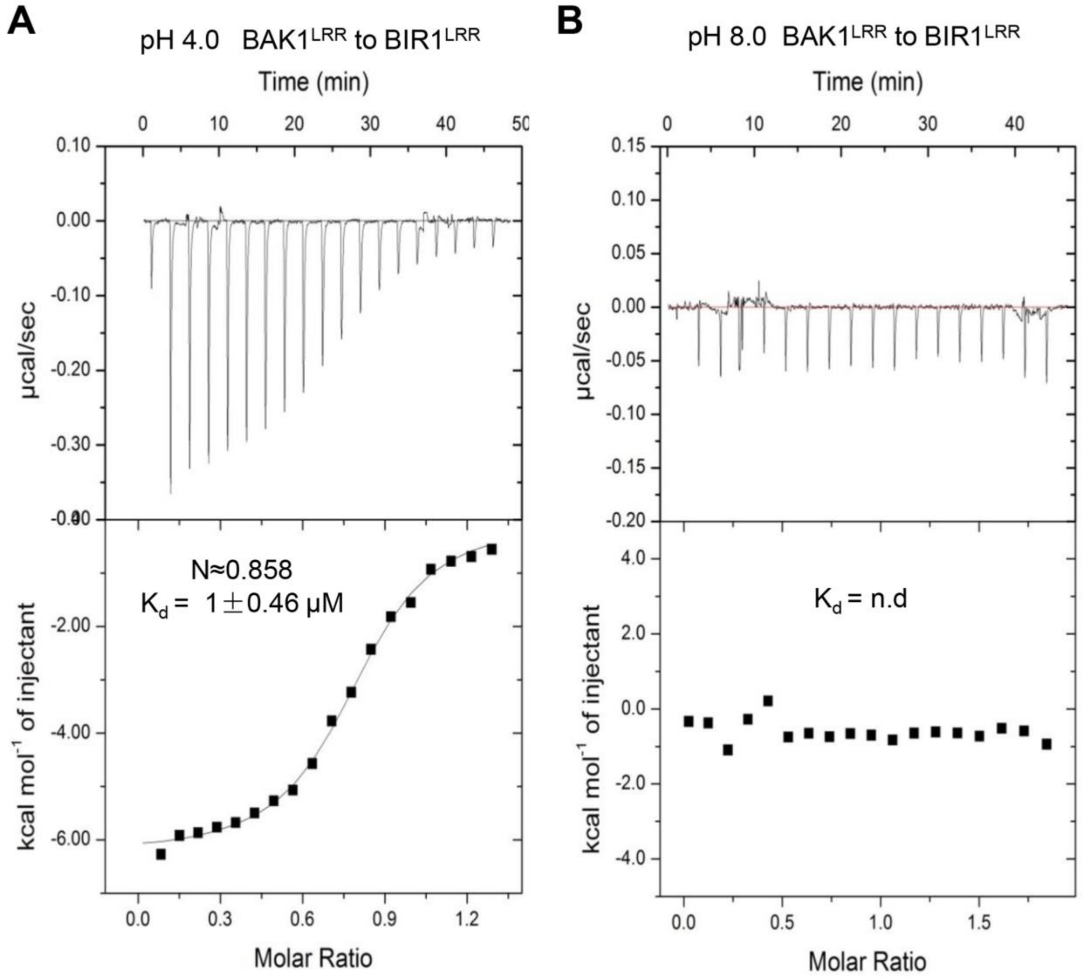

Supplementary information, Figure S4 The interaction between $BAK1^{LRR}$ and $BIR1^{LRR}$ by ITC at pH 4.0 and pH 8.0.

The assays were performed as described in Supplementary information, Figure S2A, except that buffer of pH 4.0 and pH 8.0 were used.

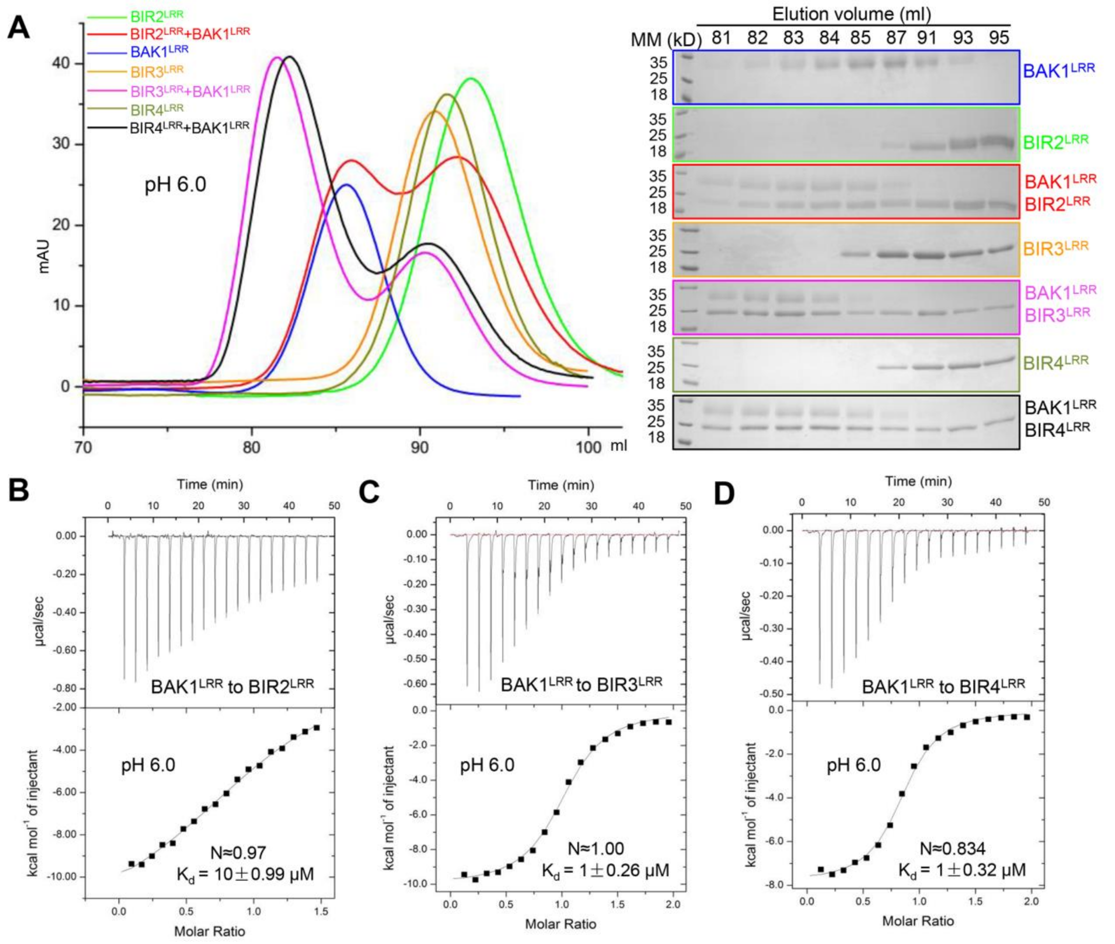

Supplementary information, Figure S5 Association of BIR2^{LRR}, BIR3^{LRR}, and BIR4^{LRR} with BAK1^{LRR} *in vitro* at pH 6.0.

(A) Gel filtration profiles of BAK1^{LRR} and BIR2^{LRR}, BIR3^{LRR} or BIR4^{LRR} at pH 6.0. The assays were performed as described in Figure 1**A**.

(B), (**C**) and (**D**) Measurement of binding affinity between BAK1^{LRR} and BIR2^{LRR} (**B**), BIR3^{LRR} (**C**), or BIR4^{LRR} (**D**) by ITC at pH 6.0. The assays were performed as described in Figure S2A. Nineteen injections of BAK1^{LRR} solution were titrated into BIR2^{LRR}, BIR3^{LRR}, or BIR4^{LRR} solution in the ITC cell. Binding affinities are indicated.

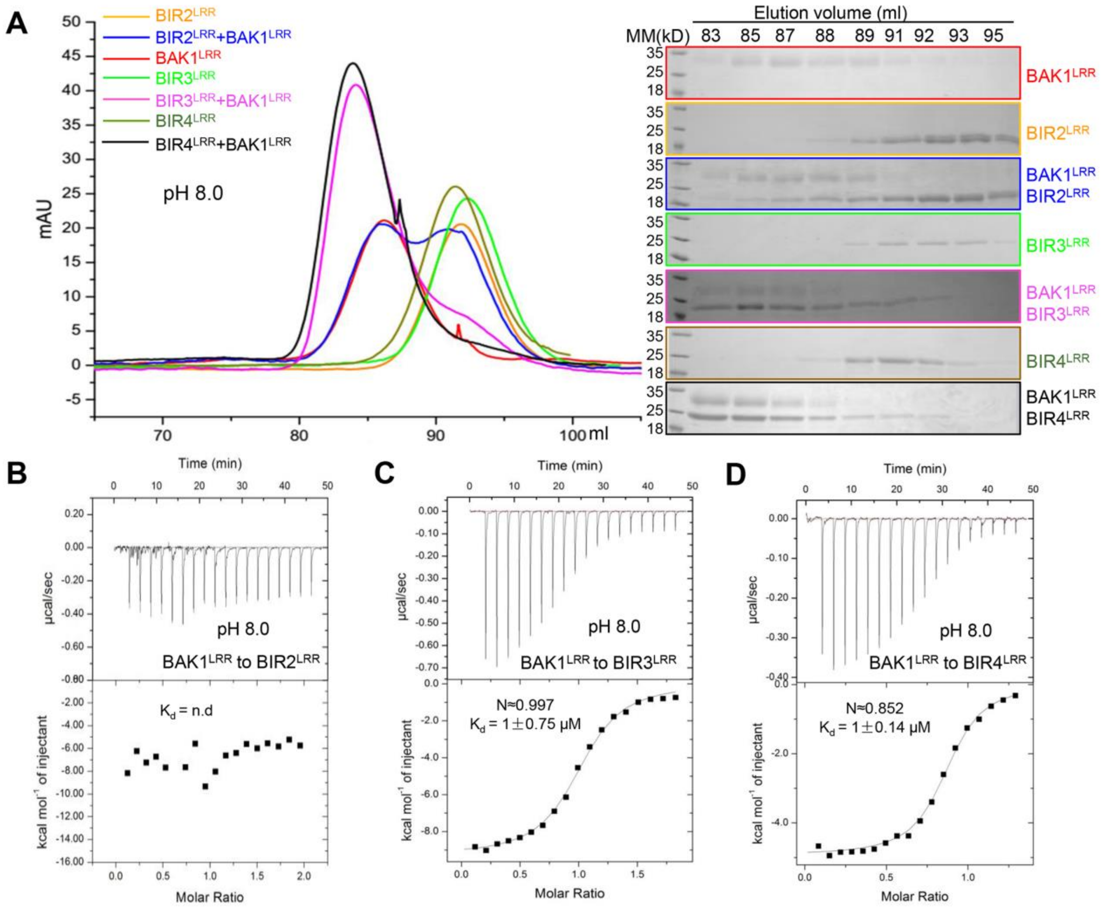

Supplementary information, Figure S6 Interaction of $BIR2^{LRR}$, $BIR3^{LRR}$, and $BIR4^{LRR}$ with $BAK1^{LRR}$ *in vitro* at pH 8.0.

(A) Gel filtration profiles of $BAK1^{LRR}$ and $BIR2^{LRR}$, $BIR3^{LRR}$ or $BIR4^{LRR}$ at pH 8.0. The assays were performed as described in Figure 1**A**.

(B) , (**C**) and (**D**), measurement of binding affinity between $BAK1^{LRR}$ and $BIR2^{LRR}$ (**B**), $BIR3^{LRR}$ (**C**), or $BIR4^{LRR}$ (**D**) by ITC at pH 8.0. The assays were performed as described in Figure S2A. Nineteen injections of $BAK1^{LRR}$ solution were titrated into $BIR2^{LRR}$, $BIR3^{LRR}$, or $BIR4^{LRR}$ solution in the ITC cell. Binding affinities are indicated. n.d: no detectable binding.

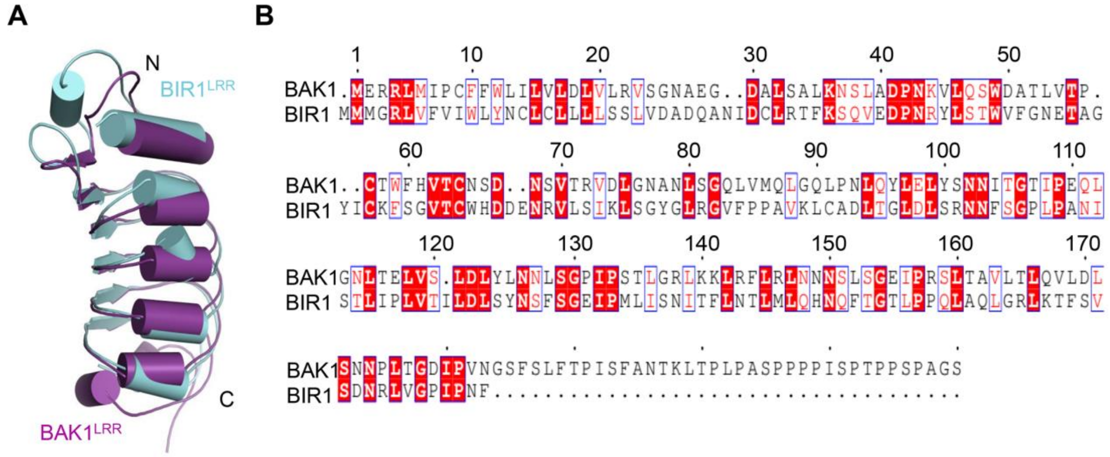

Supplementary information, Figure S7 Structure alignment and primary sequence alignment of BIR1^{LRR} and BAK1^{LRR}.

(A) BIR1^{LRR} and BAK1^{LRR} structure alignment. BIR1^{LRR} and BAK1^{LRR} have strikingly different loop regions in their conformations. “N” and “C” represent the N- and C-terminus, respectively. Color codes are indicated. This alignment was performed by the program COOT.

(B) BIR1^{LRR} and BAK1^{LRR} primary sequence alignment. Conserved and similar residues are boxed with red ground and red font, respectively.

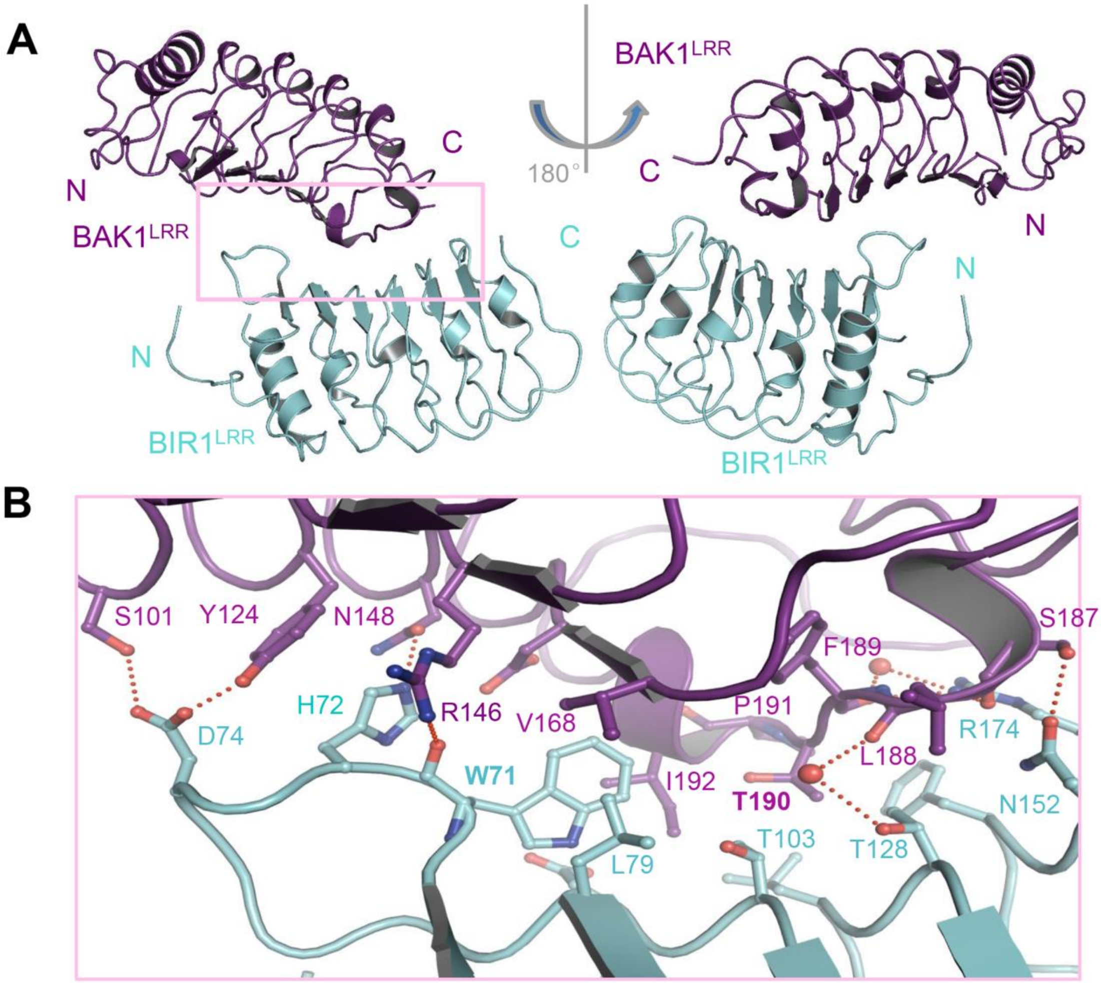

Supplementary information, Figure S8 Structural mechanism of the BAK1^{LRR} and BIR1^{LRR} interaction.

Detail interactions between BAK1^{LRR} and BIR1^{LRR} shown in an orientation different from those shown in Figure 1**C** and 1**D**. Red dashed lines indicate polar interactions. The highlighted residues are ones that generated striking phenotypes *in planta* when mutated. T, Thr; L, Leu; N, Asn; S, Ser; Y, Tyr; D, Asp; H, His; R, Arg; W, Trp; I, Ile; F, Phe.

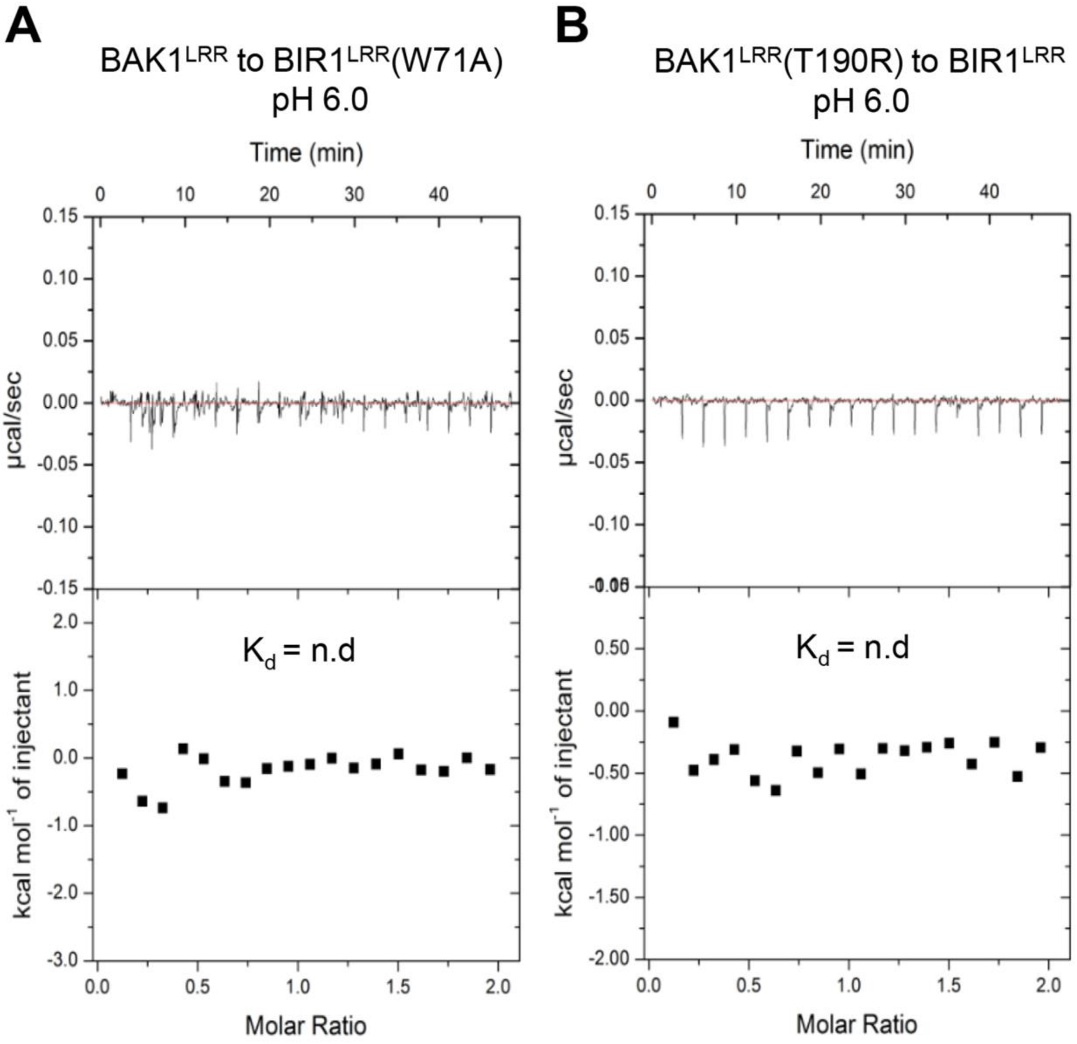

Supplementary information, Figure S9 Measurement of binding affinity between BAK1^{LRR} and BIR1^{LRR} (W71A) (**A**), and BAK1^{LRR} (T190R) and BIR1^{LRR} (**B**), by ITC at pH 6.0. The assays were performed as described in Figure **S2A**. n.d: no detectable binding.

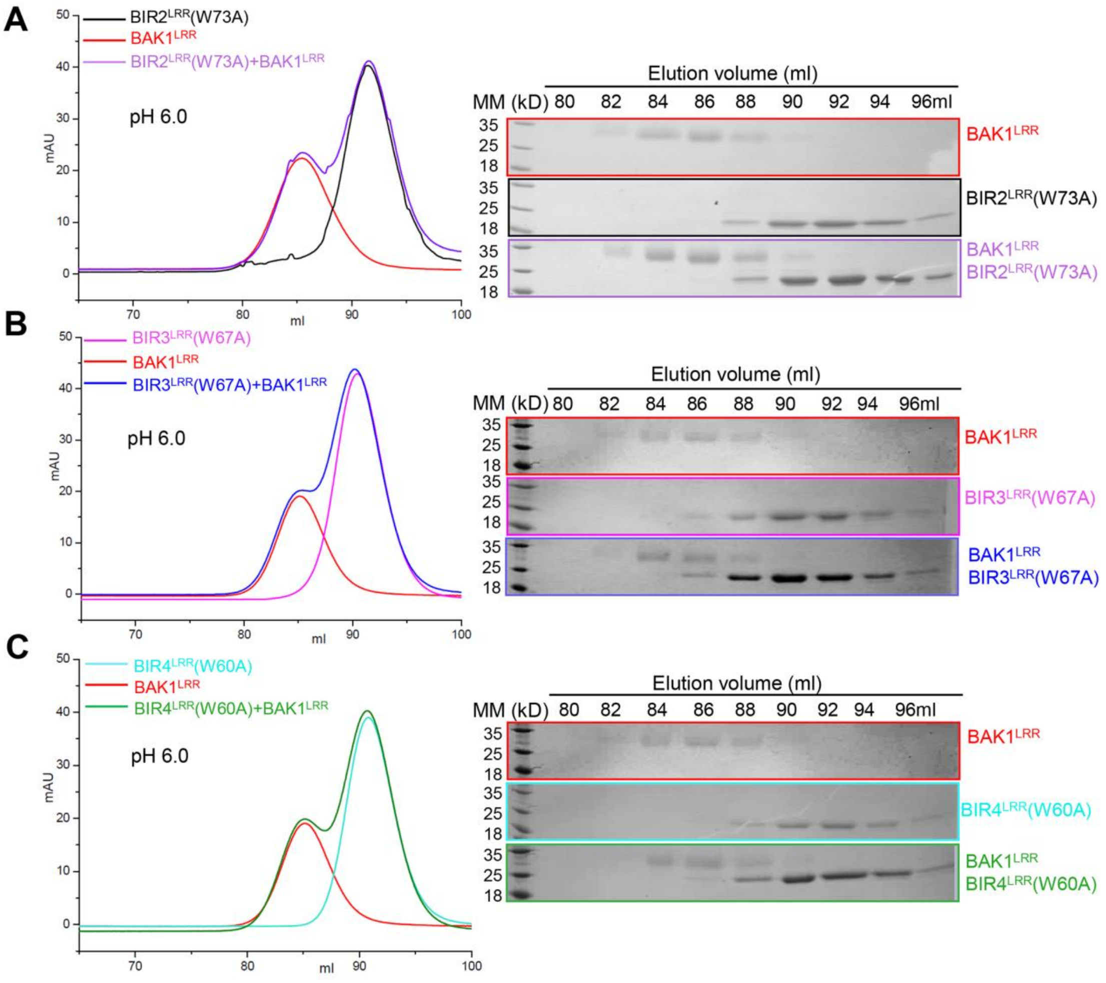

Supplementary information, Figure S10 Gel filtration profiles of BAK1^{LRR} and BIR2^{LRR} (W73A), BAK1^{LRR} and BIR3^{LRR} (W67A), or BAK1^{LRR} and BIR4^{LRR} (W60A) at pH 6.0. The assays were performed as described in Figure 1**A**.

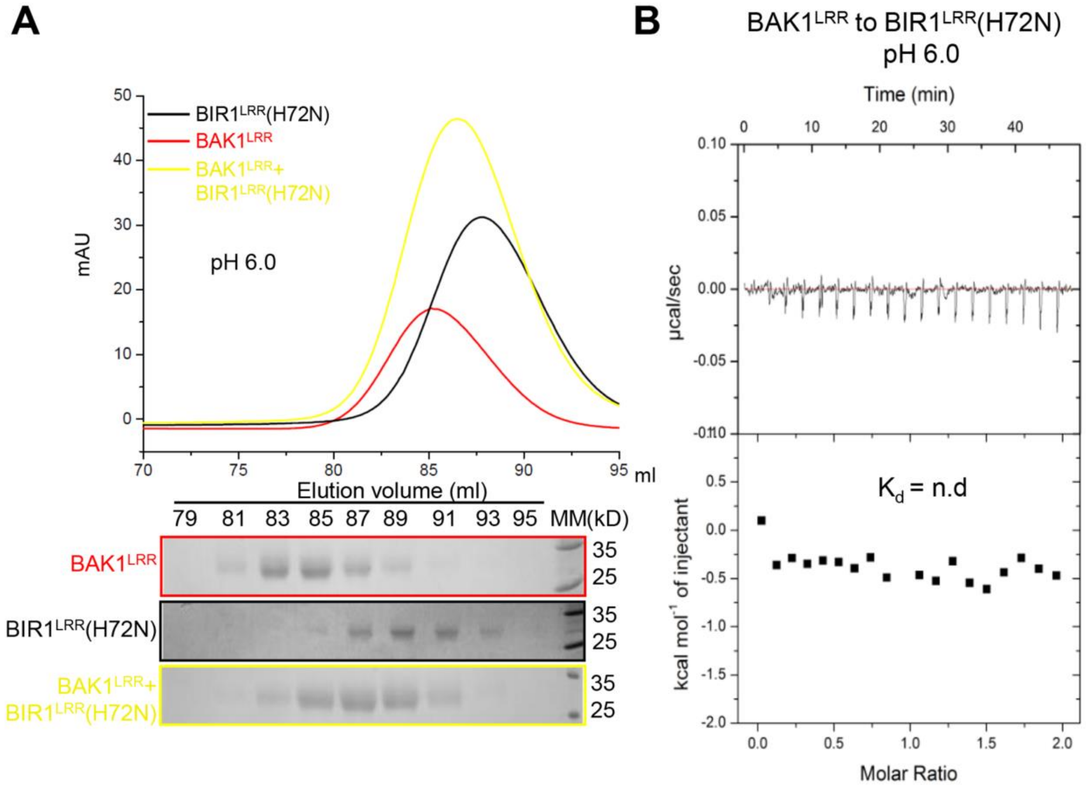

Supplementary information, Figure S11 The interaction between BAK1^{LRR} and BIR1^{LRR} (H72N) by gel filtration and ITC at pH 6.0.

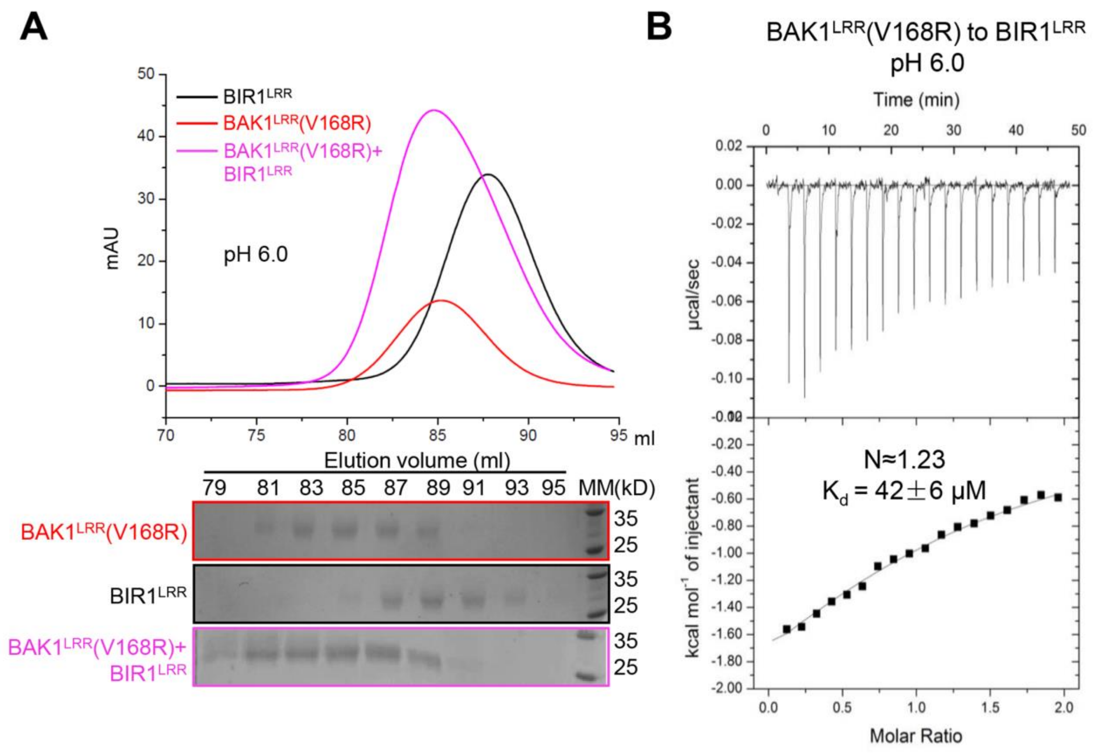

Supplementary information, Figure S12 The interaction between BAK1^{LRR} (V168R) and BIR1^{LRR} by gel filtration and ITC at pH 6.0.

On Figure **S**11 and Figure **S**12, the assays were performed as described in Figure 1**A** and **S**2**A**.

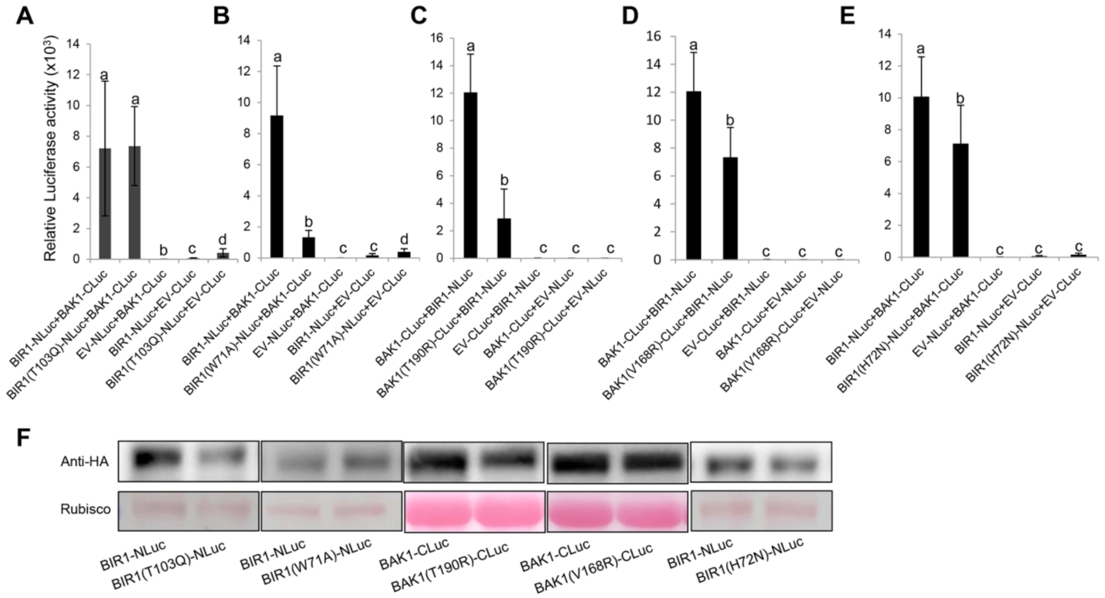

Supplementary information, Figure S13 Analysis of the interaction between BAK1 and BIR1 mutants, or BIR1 and BAK1 mutants using luciferase activity assays.

(**A**)-(**E**), quantification of LUC activity in *N. benthamiana* leaves expressing indicated proteins. Data were collected two days after infiltration. Error bars represent standard deviations of eight repeats. Statistical differences among the samples are labeled with different letters ($P < 0.01$, One-way ANOVA). The experiments were repeated three with similar results.

(**F**) The expression levels of the indicated fusion proteins in *N. benthamiana* leaves were detected using an anti-HA antibody.

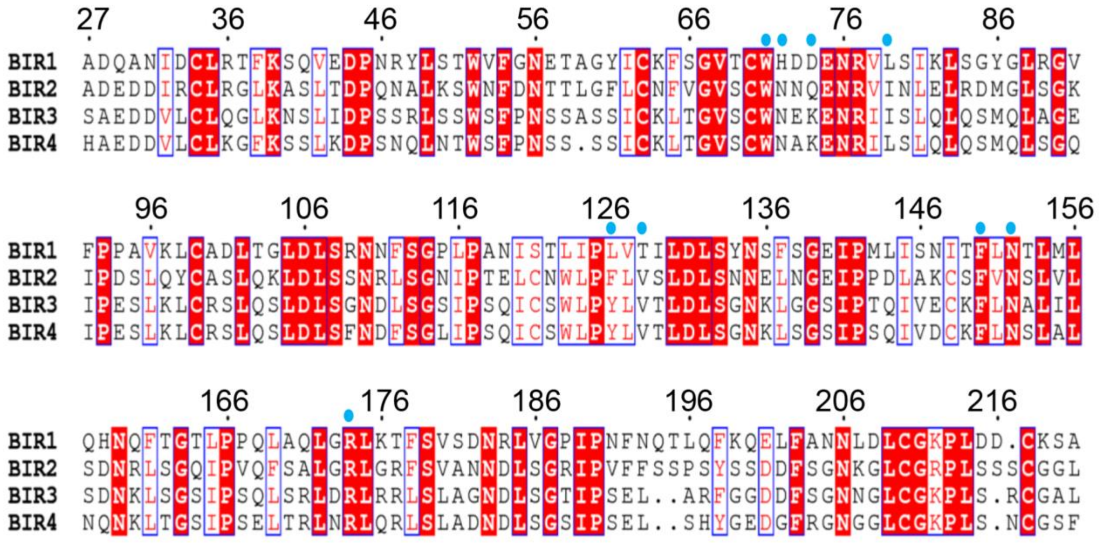

Supplementary information, Figure S14 Sequence alignment of the ectodomains of BIR family members.

Conserved and similar residues are boxed with red ground and red font, respectively. Residues involved in interaction with BAK1 are indicated with blue solid circles at the top. BAK1-interacting residues are conserved among the BIR family members.

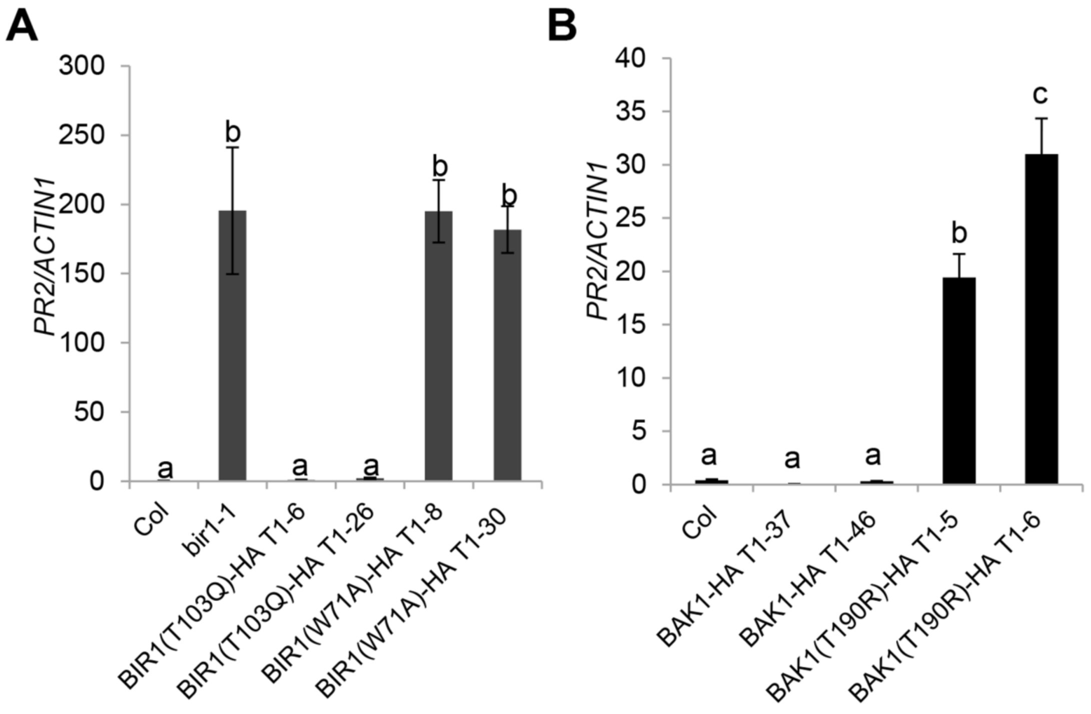

Supplementary information, Figure S15 Expression levels of *PR2* of transgenic plants expressing the BIR1 (T103Q) –HA, BIR1 (W71A) –HA, and BAK1 (T190R) –HA protein. The BIR1 (T103Q) –HA was a positive control. The assays were performed as described in Figure 1**H**.

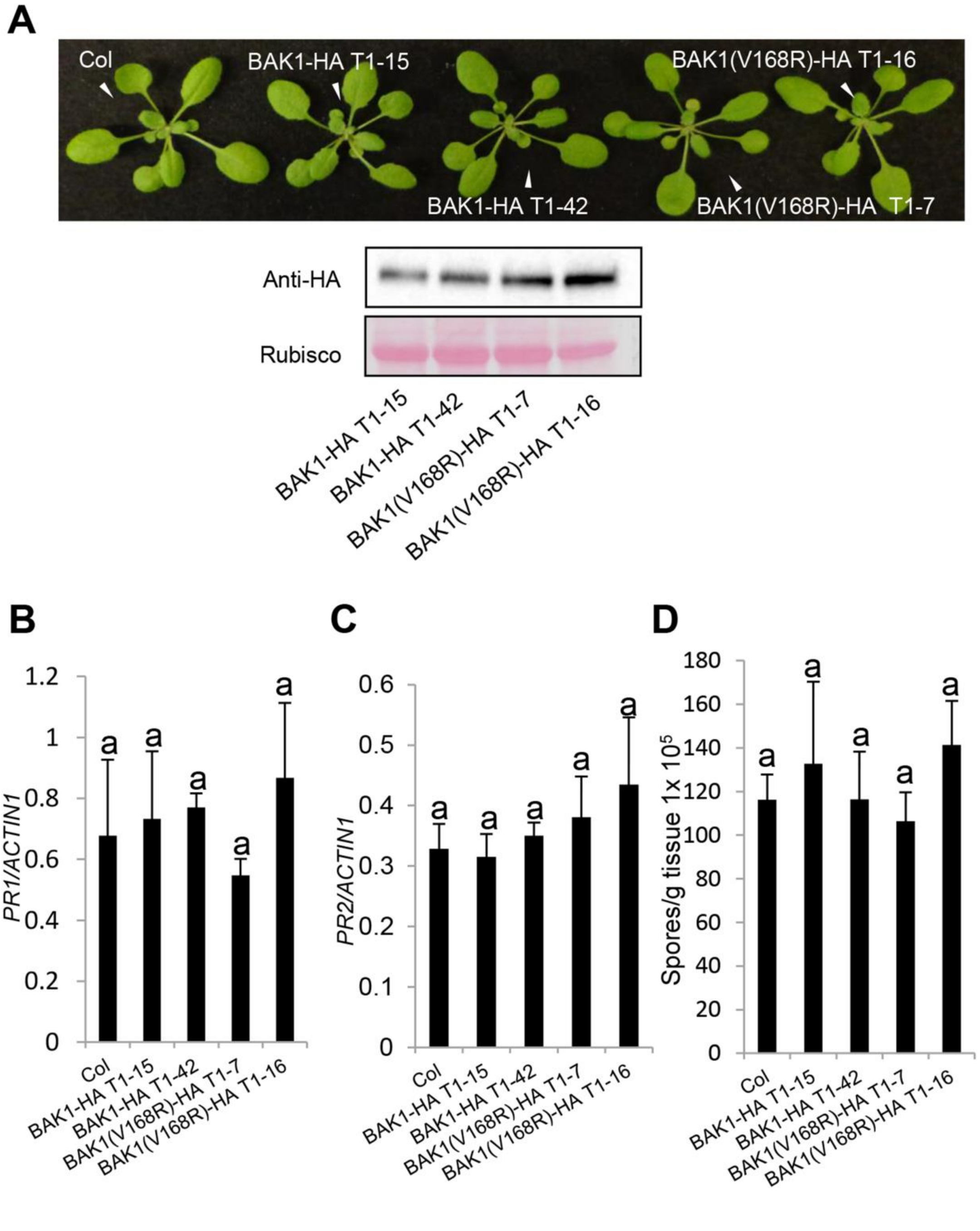

Supplementary information, Figure S16 Characterization of transgenic plants expressing the BAK1 (V168R) -HA protein.

(A) Morphological phenotypes of transgenic plants expressing the BAK1-HA or BAK1 (V168R)-HA protein under its native promoter in wild type background (Col-0).

(B) and (**C**) Expression levels of *PR1* (**B**) and *PR2* (**C**) in the indicated genotypes as determined by quantitative RT-PCR.

(**D**) Growth of *H. a.* Noco2 on the indicated genotypes seedlings.

(**A**)-(**D**), the assays were performed as described in Figure 1**G**, 1**H**, 1**J**, respectively.

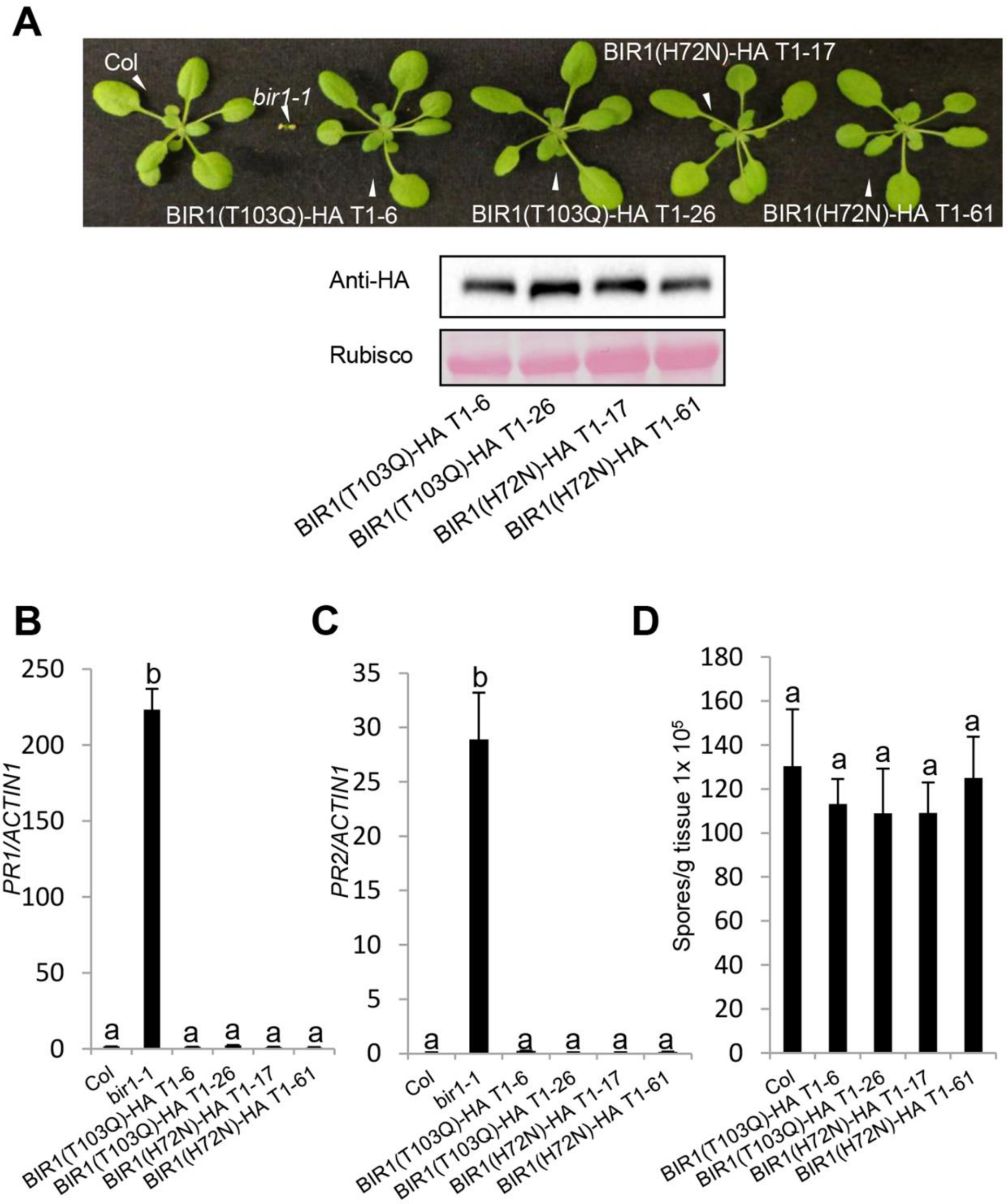

Supplementary information, Figure S17 Characterization of transgenic plants expressing the BIR1 (H72N) -HA protein.

(**A**) Morphological phenotypes of transgenic plants expressing the BIR1-HA, BIR1 (T103Q)-HA, or BIR1 (H72N)-HA protein under its native promoter in *bir1-1*.

(**B**) and (**C**) Expression levels of *PR1* (**B**) and *PR2* (**C**) in the indicated genotypes as determined by quantitative RT-PCR.

(**D**) Growth of *H. a.* Noco2 on the indicated genotypes seedlings.

(**A**)-(**D**), the assays were performed as described in Figure 1**G**, 1**H**, 1**J**, respectively.

Supplementary information, Data S1 Materials and Methods

Protein expression and purification

The LRR domains of Arabidopsis BIR1 (residues 1-226; $BIR1^{LRR}$), Arabidopsis BIR2 (residues 1-228; $BIR2^{LRR}$), Arabidopsis BIR3 (residues 1-222; $BIR3^{LRR}$), Arabidopsis BIR4 (residues 1-214; $BIR4^{LRR}$), and Arabidopsis BAK1 (residues 1-220; $BAK1^{LRR}$) with a C-terminal 6×His tag were cloned into the pFastBacTM 1 vector (Invitrogen). And the clones were identified by sequencing. $BIR1^{LRR}$ and $BAK1^{LRR}$ were expressed in high five cells at 22 °C by the Bac-to-Bac baculovirus expression system (Invitrogen), respectively. One litre of cells (2.0×10^6 cells/ml cultured in the medium from Expression System) was infected with 20 ml baculovirus, and was harvested for secreted proteins after 48 h. The proteins were purified by using Ni-NTA columns (Novagen) and then by gel filtration chromatography (Hiload 200, GE Healthcare) in buffer containing 10 mM Bis-Tris pH 6.0, 100 mM NaCl. The expression and purification procedures of all the mutants were similar to those of $BIR1^{LRR}$.

Crystallization, data collection, structure determination and refinement

The purified $BAK1^{LRR}$ and $BIR1^{LRR}$ were mixed and incubated at 4 °C for 30 min with a molar ratio of about 1:1. Then the incubated protein mixtures were concentrated to about 7.0 mg/ml for crystallization. Crystallization experiments were performed with hanging-drop vapour-diffusion methods by mixing equal volumes (1.0 μl) of protein and reservoir solution at 18 °C. About one month later, several crystals were obtained, but the crystal diffracted X-ray so poorly. To improve the diffraction ability of the crystals, BAK1 (residues 1-220;

$BAK1^{LRR}$) was changed to BAK1 (residues 1-205; $BAK1^{LRR}$). And then the incubated proteins were digested with endoglycosidase F1 and F3 at 18 °C overnight and further cleaned using gel filtration in buffer containing 100mM citric acid pH 4.0 and 100 mM NaCl. About 3 months later, good quality crystals of BAK1 (residues 1-205; $BAK1^{LRR}$) and BIR1 (residues 1-226; $BIR1^{LRR}$) were obtained in buffer containing 100 mM citric acid pH 5.0, 18% PEG 20000.

The diffraction data were collected on beam line BL17U1 using a CCD detector at the Shanghai Synchrotron Radiation Facility (SSRF). The data were processed using HKL2000[1]. The crystal structure of $BAK1^{LRR}$-$BIR1^{LRR}$ was determined by molecular replacement (MR) method with PHASER[2] and refined with PHENIX[3] using the structure of BAK1 (PDB code: 4MN8) as the initial searching model. And the model from MR was built with the program COOT and refinement by the program Phenix. The structure of $BAK1^{LRR}$-$BIR1^{LRR}$ was finally refined to a resolution of 2.3 Å with R_{work} 19.2% and R_{free} 21.5%, respectively. All the figures representing structures were prepared using PYMOL （DeLano, W. L. PyMOL Molecular Viewer. http://www.pymol.org, 2002).

Gel filtration assay

The purified $BAK1^{LRR}$ with $BIR1^{LRR}$ proteins were subjected to gel filtration analysis (Hiload 200, GE Healthcare). The $BAK1^{LRR}$ and $BIR1^{LRR}$ proteins were mixed with a molar ratio of about 1:2, and incubated at 4 °C for 30 min before the gel filtration analysis in buffer containing 10 mM Bis-Tris pH 6.0, 100 mM NaCl. The samples from the fractions were used

to SDS–PAGE and visualized by Coomassie blue staining. The BAK1^{LRR} mutants and BIR1^{LRR} mutants were designed to disrupt their interaction, and were also identified with the gel filtration assay described above.

Analysis of transgenic plants expressing BIR1 and BAK1 mutant proteins

Arabidopsis plants were grown under 16 h light at 23 °C and 8 h dark at 19 °C. PCR-based site directed mutagenesis was conducted to generate constructs expressing mutant *BAK1(T190R)-HA* in pGreenII or *BIR1(T103Q)-HA* and *BIR1(W71A)-HA* in pCambia1305. The constructs were transformed into *Agrobacterium* strain *GV3101* by electroporation. The *bir1-1* mutant was transformed with constructs expressing *BIR1 (W71A)-HA* or *BIR1 (T103Q)-HA* under *BIR1* native promoter and the Col-0 plant was transformed with constructs expressing *BAK1 (T190R)-HA* under *BAK1* native promoter using the floral dip approach.

For gene expression analysis, total RNA was extracted from two-week-old seedlings grown on soil using the EZ-10 Spin Column Plant RNA Mini-Preps Kit (Bio Basic Inc.). Reverse transcription was conducted with M-MuLV reverse transcriptase (Applied Biological Materials Inc) and quantitative PCR was carried out using the SYBR Premix Ex TaqII kit (Takara Inc.). The primers used to amplify *PR1*, *PR2* and *ACTIN1* have been described previously[4].

The pathogen infection assays using *Hyaloperonospora arabidopsidis* (*H.a.*) Noco2 were performed as previously described[5]. Two-week-old soil grown seedlings were

spray-inoculated with *H.a.* Noco2 spores (5×10^4 spores/mL) re-suspended in water. The plants were kept at 18 °C with about 80% humidity for seven days before data collection. Growth of the pathogen was measured by counting the number of conidia spores using a hemocytometer.

Split Luciferase complementation assay

Luciferase complementation assays were carried as previously described[6]. Specifically, Agrobacteria harboring constructs expressing the wild-type or mutant BAK1-CLuc and BIR1-NLuc along with the negative controls were grown overnight in LB medium and transferred to new LB medium with 50 mM acetosyringone for 6 h until OD600 reached 0.8 to 1.0. Bacteria were re-suspended into solution containing 10 mM MES pH 5.6, 10 mM $MgCl_2$, and 150 mM acetosyringone. The diluted bacteria solution (OD600=0.3) was then infiltrated into the leaves of four-week-old N. benthamiana plants. Plants were kept at 23 °C for three days before measuring luciferase activities. To quantify the luciferase activity, 50 μL luciferin solution containing 1 mM luciferin, 10 mM $MgCl_2$ and 10 mM MES/KOH buffer pH 5.6 was aliquoted into a 96-well white plate. Leaf discs from infiltrated tissue were excised using a paper punch (diameter=0.314 mm) and transferred into the wells containing the luciferin solution. The luminescence was measured using a BioTek Synergy II microplate reader.

Sedimentation-velocity analytical ultracentrifugation

Sedimentation velocity was performed with an XL-I analytical ultracentrifuge (Beckman Coulter) equipped with a four-cell An-60 Ti rotor for interaction analysis of $BAK1^{LRR}$ and $BIR1^{LRR}$ at 20 °C. On Figure S2B, the molar ratio of the complex of $BAK1^{LRR}$ and $BIR1^{LRR}$ is about 4:3, and the total OD280 is about 0.7, and the OD280 of single $BAK1^{LRR}$ is about 0.8, and the OD280 of single $BIR1^{LRR}$ is about 0.6. Buffer containing 10 mM Bis-Tris pH 6.0, 100 mM NaCl was used as the reference solution. All samples were applied at a speed of 60,000 rpm. Absorbance scans were taken at 280 nm at the intervals of 0.003 cm size in a radical direction. The different sedimentation coefficients, c(s), and molecular weight were calculated by SEDFIT V14.4f software.

ITC assay

The binding affinities of $BAK1^{LRR}$ and mutant $BIR1^{LRR}$, or mutant $BAK1^{LRR}$ and $BIR1^{LRR}$, or $BAK1^{LRR}$ and $BIR2^{LRR}$, $BIR3^{LRR}$ or $BIR4^{LRR}$ at pH 4.0, 6.0, or 8.0 were measured using MicroCalorimeter ITC200 (Microcal LLC) at 25 °C in the buffer containing 10 mM citric acid, pH 4.0, and 100 mM NaCl, or 10 mM Bis-Tris, pH 6.0, and 100 mM NaCl, or 10 mM Tris, pH 8.0, and 100 mM NaCl. Approximately 0.3 mM $BAK1^{LRR}$, or a mutant $BAK1^{LRR}$ was injected into the stirred calorimeter cell (250 µl) containing $BIR1^{LRR}$, mutant $BIR1^{LRR}$, $BIR2^{LRR}$, $BIR3^{LRR}$, or $BIR4^{LRR}$ (0.05 mM) with 1 × 0.5 µl + 18 × 2 µl at 2.5-min intervals. The stirring speed was 750 rpm. The heat of dilution obtained by the titration of peptides into

the buffer was subtracted. Each experiment was repeated at least three times. All the titration data were analyzed using the ORIGIN software (MicroCal Software).

Gel filtration analysis of $BAK1^{LRR}$-$BIR1^{LRR}$ in the presence of $FLS2^{LRR}$-flg22

The $BAK1^{LRR}$ (about 0.12 mg) and $BIR1^{LRR}$ (about 0.3 mg) proteins were mixed with a molar ratio of about 2:5, and incubated at 4 °C for 30 min. The $FLS2^{LRR}$ (about 0.96mg) and excess flg22 (synthesized by Scilight Biotechnology, China) were mixed and incubated at 4 °C for 30 min. Then half of the pre-incubated $BAK1^{LRR}$-$BIR1^{LRR}$ and half of the pre-incubated $FLS2^{LRR}$-flg22 were mixed and incubated at 4 °C for 30 min. Then the mixture was assayed using gel filtration (Hiload 200) in buffer containing 10 mM Bis-Tris pH 6.0, 100 mM NaCl. The other half of the pre-incubated $BAK1^{LRR}$-$BIR1^{LRR}$ or $FLS2^{LRR}$-flg22 was individually assayed using a similar method. The samples from the peak fractions were used for SDS–PAGE analysis and visualized by Coomassie blue staining.

References

1 Otwinowski Z, Minor W. Processing of X-ray diffraction data collected in oscillation mode. Methods in enzymology 1997; 276:307-326.
2 McCoy AJ, Grosse-Kunstleve RW, Adams PD, Winn MD, Storoni LC, Read RJ. Phaser crystallographic software. Journal of applied crystallography 2007; 40:658-674.
3 Emsley P, Cowtan K. Coot: model-building tools for molecular graphics. Acta crystallographica Section D, Biological crystallography 2004; 60:2126-2132.
4 Zhang Y, Tessaro MJ, Lassner M, Li X. Knockout analysis of Arabidopsis transcription factors TGA2, TGA5, and TGA6 reveals their redundant and essential roles in systemic acquired resistance. The Plant cell 2003; 15:2647-2653.

5 Bi D, Cheng YT, Li X, Zhang Y. Activation of plant immune responses by a gain-of-function mutation in an atypical receptor-like kinase. Plant physiology 2010; 153:1771-1779.
6 Smirnova DV, Ugarova NN. Firefly Luciferase-based Fusion Proteins and their Applications in Bioanalysis. Photochemistry and photobiology 2017; 93:436-447.
